# *Solidago canadensis* L. Herb Extract, Its Amino Acids Preparations and 3D-Printed Dosage Forms: Phytochemical, Technological, Molecular Docking and Pharmacological Research

**DOI:** 10.3390/pharmaceutics17040407

**Published:** 2025-03-24

**Authors:** Oleh Koshovyi, Yurii Hrytsyk, Lina Perekhoda, Marharyta Suleiman, Valdas Jakštas, Vaidotas Žvikas, Lyubov Grytsyk, Oksana Yurchyshyn, Jyrki Heinämäki, Ain Raal

**Affiliations:** 1 Institute of Pharmacy, Faculty of Medicine, University of Tartu, Nooruse 1, 50411 Tartu, Estonia; jyrki.heinamaki@ut.ee (J.H.); ain.raal@ut.ee (A.R.); 2 Department of Pharmacognosy and Nurtricioligy, National University of Pharmacy, 61002 Kharkiv, Ukraine; 3 Department of Pharmaceutical Management, Drug Technology and Pharmacognosy, Ivano-Frankivsk National Medical University, 76018 Ivano-Frankivsk, Ukraine; ygritsik@gmail.com; 4 Department of Medicinal Chemistry, National University of Pharmacy, 61002 Kharkiv, Ukraine; lina_perekhoda@nuph.edu.ua (L.P.); suleiman.nfau@outlook.com (M.S.); 5 Institute of Pharmaceutical Technologies, Lithuanian University of Health Sciences, 44307 Kaunas, Lithuania; valdas.jakstas@lsmu.lt (V.J.); vaidotas.zvikas@lsmu.lt (V.Ž.); 6 Department of Chemistry, Pharmaceutical Analysis and Postgraduate Education, Ivano-Frankivsk National Medical University, 76018 Ivano-Frankivsk, Ukraine; grycyk_l@ukr.net; 7 Department of Microbiology, Virology and Immunology, Ivano-Frankivsk National Medical University, 76018 Ivano-Frankivsk, Ukraine; oiurchyshyn@ifnmu.edu.ua

**Keywords:** Canadian goldenrod, modified dry extract, phenolics, amino acid, molecular docking, antibacterial effect, anti-inflammatory activity, hepatoprotective activity, 3D printing

## Abstract

**Background/Objectives**: The Canadian goldenrod (*Solidago canadensis* L.) is one of the most widespread species of the genus *Solidago* from the Asteraceae family. It has a rich composition of biologically active compounds and is traditionally used to address kidney, urinary tract, and liver diseases. Previously, it was proven that the *S. canadensis* extract obtained with a 40% ethanol solution had the most promising anti-inflammatory and hepatoprotective activity. Therefore, this extract was selected for the further formulation of amino acid preparations and 3D-printed dosage forms. The aims of the present study were to investigate the chemical composition, toxicity, and antimicrobial, anti-inflammatory, and hepatoprotective activity of *S. canadensis* dry extract, its amino acid preparations, and 3D-printed dosage forms. **Results**: A total of 18 phenolic compounds and 14 amino acids were determined in the extracts. The *S. canadensis* herb extracts were verified to be practically non-toxic preparations (toxicity class V, LD₅₀ > 5000 mg/kg). They also showed moderate antimicrobial activity against *Staphylococcus aureus*, *Enterococcus faecalis,* and β-hemolytic *Streptococcus pyogenes*. The most pronounced hepatoprotective activity was observed with *S. canadensis* herb extract and its amino acid preparations with phenylalanine, alanine, and lysine at a dose of 25 mg/kg body weight. The most pronounced anti-inflammatory activity was found with *S. canadensis* herb extract and its preparation with arginine. According to the calculated docking score array and the analysis of binding modes in the active sites of COX-1 and COX-2, the flavonoid fraction and caffeic acid in the *S. canadensis* extracts presented moderate inhibitory activity. **Conclusions**: The development of innovative 3D-printed oral dosage forms represents a promising strategy to formulate dietary supplements or pharmaceutical preparations for these herbal extracts.

## 1. Introduction

Canadian goldenrod (*Solidago canadensis* L.) is one of the most widespread species of the genus *Solidago* from the Asteraceae family. This plant, which was first introduced in the 17th century in Europe as an ornamental species, is now considered one of the most aggressive invasive species in Europe, China, and other regions [1,2]. Its ability to spread rapidly and form monodominant communities threatens biodiversity by reducing native species populations and altering ecosystem functions. The rapid expansion of *S. canadensis* is driven by mechanisms such as allelopathy and high seed productivity. Additionally, biologically active compounds in its roots significantly inhibit the germination and growth of other plant species [1,2]. The successful invasion of *S. canadensis* is further facilitated by its ability to alter soil structure and degrade soil quality by depleting nutrients [2]. Today, it is found globally due to its rapid expansion. It thrives in various soil conditions but grows best in nutrient-rich, moderately moist, heavier soils. The plant is easy to cultivate, thus ensuring a substantial raw material base. At the same time, this plant also provides certain ecosystem benefits, such as supporting pollinators through abundant nectar production [2,3]. *S. canadensis* is also an important resource for the bioeconomy, as it is used in producing natural pesticides, dyes, pharmaceutical products, and even biofuel [2,4]. Given its long history of successful use in folk medicine across multiple countries, further scientific exploration is warranted, and its broader application in conventional medicine holds promise.

*S. canadensis* is characterised by a rich composition of biologically active compounds, such as flavonoids (rutin, quercetin, kaempferol), phenolic acids (chlorogenic, caffeic), terpenoids, saponins, and essential oil [3,4,5]. According to the literature, these groups of bioactive compounds give *S. canadensis* its anti-inflammatory, antioxidant, diuretic, spasmolytic, antibacterial, and antitumor activities [3,6,7]. The phytotherapeutic uses of *S. canadensis* include the treatment of chronic nephritis, cystitis, urolithiasis, and rheumatism. Additionally, it is used for anti-inflammatory applications and as a mouth rinse for oral and throat inflammations [8,9,10].

Several publications have reported on the chemical composition of *S. canadensis* essential oil, identifying key compounds such as germacrene D, limonene, α-pinene, β-elemene, and bornyl acetate [11,12,13]. According to the European Pharmacopoeia, *Solidago herba* is standardised based on its flavonoid content, requiring a minimum of 0.5% and a maximum of 1.5%, calculated as hyperoside [14]. The primary flavonoids in the Solidago genus include glycosides of quercetin and kaempferol and their free aglycones [15,16,17]. The *S. canadensis* raw material is also rich in polyphenolic acids, such as gallic, chlorogenic, caffeic, ferulic, and vanillic acids [18,19], and oleanane-type triterpene saponins [3,20]. Flavonoids and hydroxycinnamic acids of *S. canadensis* are promising agents for treating the urinary tract and prostatitis.

Plants-origin medicines usually have a favourable safety profile. Therefore, plant-origin tinctures and extracts are often used to improve various drug treatment outcomes and effectiveness. Galenic remedies (such as tinctures, teas, decoctions, and liquid extracts) often face challenges, such as the lack of standardisation and low patient adherence. A promising strategy here could be the use of pharmaceutical 3D printing [21,22] for preparing novel oral dosage forms to improve the efficacy and patient compliance of plant-origin medicines. *S. canadensis* extracts are components of such complex herbal medicines as *Marelin*, *Phytolysin*, and *Prostamed*, which are available on the Ukrainian market [23]. In folk medicine, galenic remedies of *S. canadensis* are commonly used for treating urinary tract, kidney, and liver diseases. Externally, they are applied as washes and compresses for healing wounds, gum abscesses, and furunculosis [24,25]. Given its diverse applications, further research is needed to gain knowledge of its chemical profile and pharmacological properties and to find the most feasible pharmaceutical formulations and dosage forms for the extracts of the present plant. The successful formulation of 3D-printed medicinal dosage forms for plant-origin materials, such as *S. canadensis* extracts, may lead to a more rational and personalised use of plant-origin materials in medical and pharmaceutical practice.

Alterations to the biologically active compounds in plant extracts can help to amplify their effects. One commonly used approach is conjugating components in the extracts with amino acids [26,27,28]. For example, the modification of acyclovir with valine resulted in forming a new active compound—valacyclovir—which significantly boosts the systemic plasma levels of acyclovir, thus enhancing patient comfort and clinical effectiveness [29]. In developing L-lysine aescinate, β-escin (a triterpene saponin from chestnut) was combined with L-lysine [23,30]. It was demonstrated that arginine improves the bioavailability (the rate of absorption) and stability of perindopril while also reducing its side effects [31]. In another study, the tincture of *Leonurus cardiaca* L. was combined with amino acids, leading to the development of new extracts with stronger anxiolytic properties [32]. The combination of highbush blueberry (*Vaccinium corymbosum* L., Ericaceae) [33] and cranberry leaf extracts (*Vaccinium macrocarpon* Aiton, Ericaceae) [34] with arginine fostered the creation of novel active ingredients with promising hypoglycaemic and hypolipidemic effects. These examples highlight the potential of modifying *S. canadensis* extracts in the development of new active compounds. Previously, it was proven that the *S. canadensis* extract obtained with a 40% ethanol solution had the most promising anti-inflammatory and hepatoprotective activity [35]. Therefore, this ethanolic extract was selected to be modified with amino acids in the present study.

The aim of the present study was to investigate and gain knowledge of the chemical composition, toxicity, and antimicrobial, anti-inflammatory and hepatoprotective activity of *S. canadensis* dry extract, its amino acid preparations, and 3D-printed dosage forms. We also predicted the mechanism of the anti-inflammatory action of the extracts using in silico methods, such as molecular docking. In the present study, we introduced a novel combinatorial approach to promote the anti-inflammatory and hepatoprotective activity of *S. canadensis* extract by combining the extract with amino acids. The positive effect of this strategy on the anti-inflammatory and hepatoprotective activity of the extract was verified. Moreover, we developed a novel aqueous-based gel formulation for the SSE 3D printing of *S. canadensis* extract and for preparing novel oral dosage forms for the present plant extract.

## 2. Materials and Methods

### 2.1. Materials

The flowering tops of *S. canadensis* were collected in Tartu, Estonia (58.36277085085124, 26.747175570884128) in July 2023 and passed to Ukraine. The species was identified by Professor A.R. Grytsyk using keys from the botanical catalogue [36]. The raw plant material (around 1 kg) was dried (at 20 ± 2 °C) for 14 days in a well-ventilated space and stored in paper bags. Voucher specimens No. 455–457 were kept at the Department of Pharmaceutical Management, Drug Technology, and Pharmacognosy, Ivano-Frankivsk National Medical University.

For preparing the dry extracts, 500.0 g of dried *S. canadensis* herb was macerated with 1000 mL of 40% aqueous ethanol solution in an extractor at room temperature for one day. Following this, the liquid extract was separated, and the procedure was repeated twice with the new portions of the same extractant (1000 mL each). Three liquid extracts were combined and allowed to settle for two days and filtered. The yield of the dry extract was 26.46%. To modify the extract with amino acids, a total of six (6) portions of this liquid extract (400 mL each) were prepared. In selecting amino acids, we considered previous experience with similar modifications of other plant extracts [26,28,30] and chose those with the greatest potential impact. The following amino acids were added in triple equimolar amounts to the content of polyphenolic compounds: phenylalanine (OstroVit, Zambrov, Poland, 2.5786 g), L-arginine (FITS, Tallinn, Estonia, 2.7165 g), glycine (OstroVit, Zambrov, Poland, 1.1712 g), L-lysine (FITS, Tallinn, Estonia, 2.2809 g), β-alanine (OstroVit, Zambrov, Poland, 1.3900 g), and valine (Acros Organics, Geel, Belgium, 1.8227 g). These solutions with the amino acids and the remainder of the primary liquid extract were then left to infuse for 24 h, after which they were evaporated with a rotary vacuum evaporator Buchi B-300 (Buchi AG, Flawil, Switzerland) to form soft extracts, which were further freeze-dried (lyophilised) with a SCANVAC COOLSAFE 55-4 Pro freeze dryer (LaboGene ApS, Allerød, Denmark). The dry extracts prepared were referred to as S, S-Phe, S-Arg, S-Gly, S-Lys, S-Ala, and S-Val, respectively.

### 2.2. Assay of Main Phytochemicals by Spectrophotometry

The assay of main phenolic substances (hydroxycinnamic acids, flavonoids, and total phenolic compounds) in the *S. canadensis* dry extract and its amino acid preparations was carried out with a Shimadzu UV-1800 (Shimadzu Corporation, Kyoto, Japan) spectrophotometer using European Pharmacopeia methods. For hydroxycinnamic acids, it was based on a reaction with sodium molybdate and sodium nitrite equivalent to chlorogenic acid [14,19]. The content of flavonoids was determined using a reaction with aluminium chloride and rutin as a standard compound at the analytical wavelength of 417 nm [19,37]. Total phenolic compounds were quantified using gallic acid as a reference standard and measuring the absorbance at 270 nm [38,39]. Each experiment was repeated three times to ensure statistical validity.

### 2.3. Analysis of Phenolic Compounds by UPLC-MS/MS

The qualitative and quantitative assessment of phenolic compounds in the *S. canadensis* extracts was carried out with an ultra-performance liquid chromatography-tandem mass spectrometry (UPLC-MS/MS) system using an Acquity H-Class UPLC chromatograph (Waters, Milford, MA, USA). The column (YMC Triart C18 column (100 × 2.0 mm, 1.9 µm)) temperature was 40 °C. The mobile phase flow rate was 0.5 mL/min with solvent A (a 0.1% aqueous formic acid solution) and solvent B (pure acetonitrile). Gradient elution: solvent B was 5% from 0 to 1 min, increased to 30% from 1 to 5 min, further increased to 50% from 5 to 7 min, followed by column washing with solvent B from 7.5 to 8 min, and re-equilibration to the initial conditions (5% solvent B) from 8.1 to 10 min. The chemical structure of phenolics was investigated with a Xevo triple quadrupole tandem mass spectrometer (Waters, Milford, MA, USA). Negative electrospray ionisation (ESI) mode was applied to generate ions for MS/MS analysis. Its parameters were capillary voltage set to −2 kV, desolvation nitrogen gas heated to 400 °C with a flow rate of 700 L/h, gas flow maintained at 20 L/h, and ion source temperature set at 150 °C. The identification of phenolics was carried out by comparing MS/MS spectral data of standards and their retention times. The assay was performed using linear regression fit models and the standard dilution method [19,24,40].

### 2.4. Assay of Amino Acids by UPLC-MS/MS

The amino acids assay in the *S. canadensis* extracts was carried out using an Acquity H-Class UPLC system (Waters, Milford, MA, USA) equipped with a Xevo TQD mass spectrometer (Waters, Milford, MA, USA). A BEH Amide column (150 mm × 2.1 mm, 1.7 µm, Waters, Milford, MA, USA) temperature was 25 °C. A 1 µL sample was injected. The mobile phase: an aqueous solution of 10 mmol ammonium formate with 0.125% formic acid (eluent A) and acetonitrile (eluent B). The flow rate is 0.6 mL/min. Gradient elution: 95% B from 0 to 1 min, decreased to 70% B from 1 to 3.9 min, further reduced to 30% B from 3.9 to 5.1 min, followed by column flushing with 70% of eluent A from 5.1 to 6.4 min. At 6.5 min, the gradient was returned to the initial conditions for a total run time of 10 min. Settings of the mass spectrometer (in positive electrospray ionisation (ESI) mode): capillary voltage of +3.5 kV, cone voltage of 30 V, desolvation gas flow at 800 L/h, and desolvation temperature of 400 °C. The ion source temperature was 120 °C. Identification and peak assignment of amino acids in *S. canadensis* extracts were carried out by comparing MS/MS spectral data with analytical-grade standards and their retention times. Assay was achieved using linear regression fit models and the standard dilution method [19,41].

### 2.5. Molecular Docking Analysis

The Autodock 4.2 software package (Autodock, San Diego, CA, USA) was used for receptor-orientated flexible docking. Ligands were prepared using a MGL Tools 1.5.6 program (The Scripps Research Institute, San Diego, CA, USA). The Ligand optimisation was carried out using an Avogadro program (Autodock, San Diego, CA, USA). The ligand and receptor data output formats were changed to a special PDBQT format for carrying out calculations in the Autodock 4.2 program. As biological targets for docking, the identified phenolic active macromolecule centres with COX-1 (PDB ID: 3KK6) and COX-2 enzymes (PDB ID: 5JW1) were used. The data were used from the Protein Data Bank (PDB). MGL Tools and AutoGrid programs generate the receptor maps. The visual analysis of complexes was performed using a Discovery Studio Visualizer program (Dassault Systèmes, San Diego, CA, USA).

For docking, the physiologically active parts of flavonoids and their glycosides were selected—specifically, the flavonoid skeletons of quercetin, isoquercetin, rutin, isorhamnetin-3-*O*-rutinoside, and kaempferol-3-*O*-rutinoside. Chlorogenic, neochlorogenic, 4,5-dicaffeoylquinic, 3,5-dicaffeoylquinic, and 3,4-dicaffeoylquinic acids, which are present in the extracts, may undergo hydrolysis during metabolism to form the main active metabolites—caffeic and quinic acids. Therefore, these acids were also subjected to docking [42,43,44,45]. Celecoxib was selected as a reference drug since its binding sites with COX-1 (PDB ID: 3KK6) and COX-2 (PDB ID: 5JW1) are well known [46,47]. The evaluation values obtained from our redocking process (scoring function, free energy, and binding constant) for celecoxib were used as standard reference values.

### 2.6. Pharmacological Research

The hepatoprotective, anti-inflammatory activity and acute toxicity (LD50) of *S. canadensis* extract and its amino acid preparations were studied in accordance with the methodological guidelines of the State Expert Center of the Ministry of Health of Ukraine [48] at the clinical-biological experimental base of IFNMU.

All animal procedures were performed in compliance with the National “General Ethical Principles of Animal Experiments” (Ukraine, 2001), aligning with the provisions of the “European Convention for the Protection of Vertebrate Animals Used for Experimental and Other Scientific Purposes” (Strasbourg, 1986) [49,50,51]. We also consider the ethical and moral-legal principles that ensure the humane treatment of experimental animals for scientific and educational purposes (Protocol of the Ethics Committee of IFNMU No. 139/23 dated 16 November 2023).

A total of 140 white outbred rats of both sexes (weighing 130–240 g) and 48 sexually mature male mice (weighing 19–25 g) were used in the present study. The animals were bred in the nursery of the clinical-biological experimental base of IFNMU. The animals were standardised based on physiological and biochemical indicators and were kept in accordance with sanitary and hygienic norms on a standard diet. Laboratory animals were housed according to the current “Sanitary Rules for the Arrangement, Equipment, and Maintenance of Experimental-Biological Clinics (Vivariums)” at 18–20 °C and relative humidity of 50–55%. They were fed a balanced diet, following a standard regimen with free access to water.

#### 2.6.1. Acute Toxicity of the Extracts

The acute toxicity of the *S. canadensis* extract and its amino acid preparations was evaluated following the preclinical safety assessment methodology for medicinal products [48]. Mice were divided into groups, each consisting of six animals: the control group received purified water, and the groups received the dry *S. canadensis* extract and its amino acid preparations. The animals were monitored for 14 days, with the toxicity levels assessed based on changes in their general condition and mortality rates. The toxicity classification was determined according to widely accepted standards [19,48].

#### 2.6.2. Antimicrobial and Antifungal Activity of the Extracts

The antimicrobial activity of *S. canadensis* extracts was determined using the clinical isolates of microorganisms. They were identified using biochemical micro-tests, such as “ENTEROtest 24”, “STAPHYtest 16,” “STREPTOtest 16”, and “NEFERMENTtest 24” (Lachema, Czech Republic) [52]. Yeast-like fungal cultures were identified using the VITEK 2 system with the VITEK 2 YST ID card (bioMérieux, Marcy-l’Étoile, France).

For screening the antimicrobial activity of *S. canadensis* extracts, the agar diffusion micro-method developed in the Department of Microbiology, Virology, and Immunology of IFNMU was used [53,54]. This highly sensitive and discriminative method enables a reliable separation between active and inactive extracts. For analysis, 20 µL of *S. canadensis* extract (10 mg/mL) was used. Incubation time was 24 h for the bacteria and 2 and 4 days for fungi. The diameters of the growth inhibition zones were measured on digital images of the cultures using the UTHSCSA ImageTool 3.0 software (The University of Texas Health Science Center in San Antonio, ©1995-2002).

The *S. canadensis* extracts (including those with amino acids) at a 10 mg/mL concentration did not exhibit any antimicrobial activity. Therefore, they were re-examined at the concentration of 100 mg/mL. The antimicrobial effects of the extracts were evaluated against such bacterial strains as *Staphylococcus aureus*, *Enterococcus faecalis*, β-hemolytic *Streptococcus pyogenes*, α-hemolytic *Streptococcus anginosus*, *Streptococcus pneumoniae*, *Escherichia coli*, *Acinetobacter baumani*, *Pseudomonas aureginosa*, *Candida albicans*, *Candida lusitaniae*, and *Candida lipolytica*.

#### 2.6.3. Anti-Inflammatory Activity of the Extracts

The anti-inflammatory activity of the *S. canadensis* extract and its amino acid preparations was studied on 70 sexually mature, outbred white rats of both sexes (weighing 150–240 g). Acute aseptic inflammation was induced by the sub-plantar administration of a 2% formalin solution (0.1 mL) into the hind paw of the rats. An increase in paw volume indicated the development of an inflammatory response [33,48].

The substances being studied were administered to the animals intra-gastrically in the form of aqueous solutions at a dose of 100 mg/kg body weight. For comparison, the anti-inflammatory activity of the following well-known synthetic and plant-origin drugs was also studied: sodium diclofenac (“Diclofenac-Darnitsa” injection solution 25 mg/mL, 3 mL in ampoules, PrJSC “Darnitsa”, Kyiv, Ukraine) and quercetin granules (“Quercetin”, PJSC Scientific-Production Center “Borshchahivskiy Chemical and Pharmaceutical Plant”, Kyiv, Ukraine) [21]. Sodium diclofenac and quercetin were administered intra-gastrically at 8 mg/kg and 5 mg/kg body weight, respectively. The extracts and drugs were administered to the animals twice: two hours before the formalin injection and immediately after the injection.

The animals were divided into ten groups, with 7 individuals in each group. Groups 1–7 received the aqueous solutions of *S. canadensis* herb extracts; Group 8 received sodium diclofenac; Group 9 received quercetin; and Group 10 served as a control group.

The inflammatory response was assessed using an oncometric method [48]. The measurements were taken before the experiment and subsequently 1, 3, and 5 h after the formalin administration. The anti-inflammatory activity of extracts was established by the level of formalin-induced rat paw oedema inhibition compared to the control group.

#### 2.6.4. Hepatoprotective Activity of the Extracts

The hepatoprotective activity of *S. canadensis* extract and its amino acid preparations was investigated with the model of acute tetrachloromethane-induced hepatitis [19,48], since tetrachloromethane is capable of causing changes in the liver of animals at the morphological and biochemical levels.

To study hepatoprotective effects, the test animals (rats) were administered the extracts at 25 mg/kg body weight. The domestic Ukrainian hepatoprotector, silymarin (“Silibor” tablet from the pharmaceutical company “Zdorovya,” Kharkiv, Ukraine), was used as a comparison. The coating of tablets was removed, then the tablets were ground in a mortar and administered orally as a 1% starch suspension at a dose of 25 mg/kg body weight. The animals of a control group were given purified water [33,48].

Liver damage in the experimental animals (except for the intact ones) was induced by a 50% oil solution of tetrachloromethane (0.8 mL per 100 g body weight over two days with a 24-h interval), which was administered subcutaneously. The *S. canadensis* extracts and reference drug were orally administered 1 h before and 2 h after the administration of tetrachloromethane. The intact animals and control pathology group consumed just purified water [48,55].

The hepatoprotective activity of *S. canadensis* extracts was studied with 70 white outbred adult rats (weighing 130–240 g). The animals were divided into the following 10 groups (7 animals in each group): Group 1—intact animals; Group 2—the control group, the animals received the 50% oil solution of tetrachloromethane; Groups 3–9—the animals received *S. canadensis* extract and its amino acid preparations; Group 10—the animals received a reference substance (silymarin).

On the third day after the first tetrachloromethane administration, the animals were euthanised by decapitation, and blood was collected. Then, the liver was removed from the animals and weighed to calculate a liver mass index (LMI) and prepare the homogenate. The biochemical and functional indicators of liver and blood serum were used as criteria for determining the extracts’ hepatoprotective effectiveness. They were measured 24 h after the last administration of tetrachloromethane.

These biochemical parameters were studied at the Bioelementology Center of the Ivano-Frankivsk National Medical University, Ukraine. The intensity of peroxidative destructive processes in the animals was assessed by the TBA-active products (TBA-AP) content in the liver homogenate. The hepatoprotective activity of the *S. canadensis* extracts was determined by changes in the levels of alanine aminotransferase (ALT), aspartate aminotransferase (AST), and alkaline phosphatase (ALP) in the blood serum. The present enzymes are the hepatospecific markers of cytolysis. ALT, AST, and ALP activity was determined spectrophotometrically using standard reagent kits obtained from “Filisit-Diagnostics” (Dnipro, Ukraine). The level of lipid peroxidation products—TBA-AP—was evaluated spectrophotometrically using a reaction with 2-thiobarbituric acid (according to the method of E.N. Korobeynikova) [56].

### 2.7. Three-Dimensional (3D) Printing of S. canadensis Extracts

Polyethylene oxide, PEO (MW ~900,000, Sigma-Aldrich, St. Louis, MO, USA), was used at a 12% (*w*/*w*) concentration for preparing the aqueous gels containing *S. canadensis* extract for semi-solid extrusion (SSE) 3D printing. 1.2 g of PEO was dispersed in 10 mL of purified water and allowed to hydrate at room temperature for 13–15 h [34,57]. Tween 80 (Laborat GMBH, Berlin, Germany) was incorporated to enhance the stability and homogeneity of the printing gel and to facilitate the release of *S. canadensis* extract from the printed scaffolds [58]. The printing gel formulation consisted of *S. canadensis* extract in varying amounts (0.5, 1.0, and 1.5 g) and 0.5 g of Tween 80 as a surfactant. The viscosity of the printing gels was assessed at 22 ± 2 °C using a Physica MCR 101 rheometer (Anton Paar, Graz, Austria).

The 12% PEO gels loaded with the *S. canadensis* extract were directly printed using a Hyrel 3D printer (System 30 M, Hyrel 3D, Norcross, GA, USA). The printing process was controlled using Repetrel software, Rev3.083_K (Hyrel 3D, Norcross, GA, USA). The parameters set for SSE 3D printings were as follows: a printing head speed of 0.5 mm/s, a blunt needle (gauge 21G), no heating in a syringe, and a printing platform was used.

The two 3D-printed structures prepared and investigated were a 4 × 4 grid lattice (30 × 30 × 0.5 mm) and circular scaffolds with a 20 mm diameter. The present 3D-printed models were generated using Autodesk 3ds Max Design 2017 (Autodesk Inc., San Francisco, CA, USA) and FreeCAD (version 0.19, released in 2021) [59]. The 3D-printed lattices consisted of a total of six printed layers, whereas the circular scaffolds consisted of five layers. The 3D-printed preparations were allowed to dry for a short period of time on the printing platform (plate) at room temperature (22 ± 2 °C) until they were removed.

The 3D printability was investigated by determining the weight and surface area of the printed lattices. The theoretical surface area of a 3D-printed lattice was 324 mm^2^, which was compared with the surface area of the experimental 3D-printed structures [57,58]. The images of the printed objects were analysed using ImageJ software (National Institute of Health, Bethesda, MD, USA, version 1.51k). The weight of the 3D-printed lattices and circular scaffolds was determined with an analytical balance (Scaltec SBC 33, Scaltec, Germany).

### 2.8. Statistical Analysis

All experimental data were processed using variational statistics, calculating arithmetic average means and standard deviations using Student’s *t*-test (*p* ≤ 0.05) and Microsoft Excel 2007 software (Microsoft Corporation, Redmond, WA, USA) according to the requirements of the State Pharmacopoeia of Ukraine [60,61].

## 3. Results

The *S. canadensis* extracts were yellow-brown powders with a characteristic odour. After short-term storage, the extracts were combined (modified) with alanine and glycine, this treatment transformed the extracts into dark brown viscous masses.

### 3.1. Phytochemical Research

The phenolics of the dry *S. canadensis* extract and its amino acid preparations were studied by a UPLC-MS/MS (Table 1). A total of 18 compounds were determined, including 8 hydroxycinnamic acids, 8 flavonoids, and 2 phenolic acids. In addition, the content of the main group of phenolics (hydrocinnamic acids, flavonoids, and total phenolics) was assayed by spectrophotometry (Table 1).

### 3.2. Molecular Docking Research

The amino acid composition of *S. canadensis* dry extract and its amino acid preparations are presented in Table 2. The compositions were studied using UPLC-MS/MS.

The results of molecular docking studies are summarised in Table 3. The results showed that none of the molecules studied here demonstrated better or comparable values in comparison with Celecoxib.

The energetically most beneficial positions were found with the flavonoid fraction compounds (quercetin, isorhamnetin, kaempferol) and caffeic acid (average docking values).

### 3.3. Acute Toxicity Study

No fatalities were observed in any of the groups during the entire study period. After a single intragastric administration of *S. canadensis* extract and its amino acid preparations in mice, no signs of intoxication were observed on the day of administration or during the 14-day observation period. The animals remained clean, active, responsive to auditory stimuli, and responsive to visual stimuli. Urination and defecation processes were normal, no respiratory disturbances or convulsions were observed, and reflex excitability was preserved. The water and food consumption of mice in these groups remained normal. A study of body weight dynamics in all experimental groups was conducted to assess the toxic effects of *S. canadensis* herb extracts in mice, and the results are summarised in Table 4. The results showed that after a single intragastric administration of *S. canadensis* herb extracts, the body weight of the animals increased throughout the observation period. The increase in body weight was statistically significant compared to the baseline data of each group on the 14th day of the experiment.

At the end of the experiment, the animals (mice) were euthanised by decapitation under ether anaesthesia. The necropsy study involved a macroscopic examination of internal organs and the determination of their mass. Macroscopic analysis revealed no signs of pathological processes. The internal organs of animals that received *S. canadensis* extract did not differ in shape, size, colour, or consistency from those of intact animals. The serous membranes in the abdominal cavity remained unchanged. The average mass and mass variation in the internal organs in mice are shown in Table 5. The results revealed the absence of hepatotoxic and nephrotoxic effects of *S. canadensis* herb extracts after a single intragastric administration in mice.

In the toxicity study, the changes in the ALT and AST activity in blood serum and the de Ritis ratio (reflecting the AST/ALT activity ratio) were investigated in mice. The results obtained with mice on the 14th day of the study are presented in Table 6. The results revealed that a single administration of plant extracts did not exert toxic effects in mice, and the biochemical parameters of blood did not significantly differ from the corresponding data obtained with the intact mice control group.

### 3.4. Antimicrobial and Antifungal Activity

The antimicrobial and antifungal activity of *S. canadensis* (goldenrod) extracts at a concentration of 100 mg/mL was studied using an agar diffusion micro-method (Table 7).

### 3.5. Anti-Inflammatory Activity

The anti-inflammatory activity of the *S. canadensis* extract and its amino acid preparations in the rat paw formalin inflammation model is presented in Table 8 and Table 9.

### 3.6. Hepatoprotective Activity

The hepatoprotective action of *S. canadensis* extract and its amino acid preparations was investigated by determining the survival rate of the animals, LMI, and the normalisation of biochemical indicators in the blood serum and liver homogenate [48]. On the second day of the experiment, one animal died in the control group. All other animals (rats) remained alive until the end of the experiment, which demonstrates the promising potential of the present substances for hepatoprotective effects. Table 10 shows an increase in the liver mass index (LMI) of animals (rats), thus indicating liver swelling and disturbances in its circulation [48].

The effects of *S. canadensis* extracts on the levels of biochemical indicators in blood serum (ALT, AST, and ALP) and liver homogenate (thiobarbituric acid-active products, TBK-AP) in rats are presented in Table 11.

### 3.7. Novel 3D-Printed Oral Dosage Forms for S. canadensis Extract

**Figure 1:**
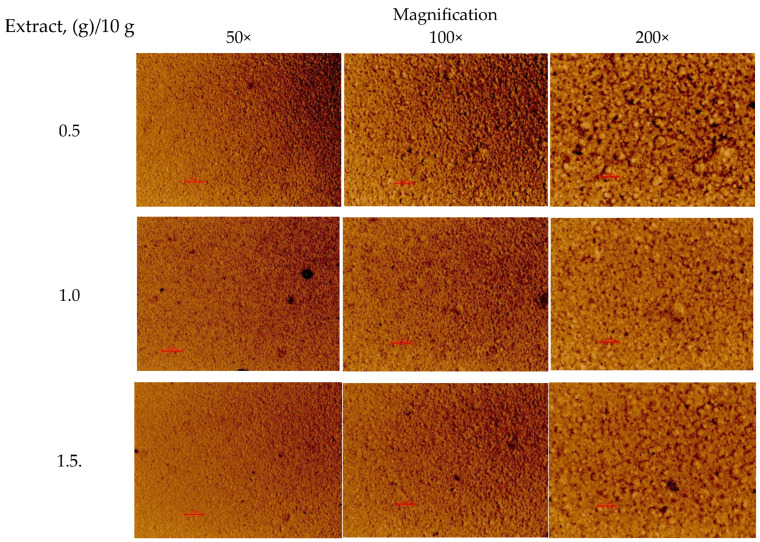
Optical light microscopy images of the polyethylene oxide (PEO) gels with the *S. canadensis* extract. Magnification 50×, 100×, and 200×. Red scale = 10 µm.

**Figure 2:**
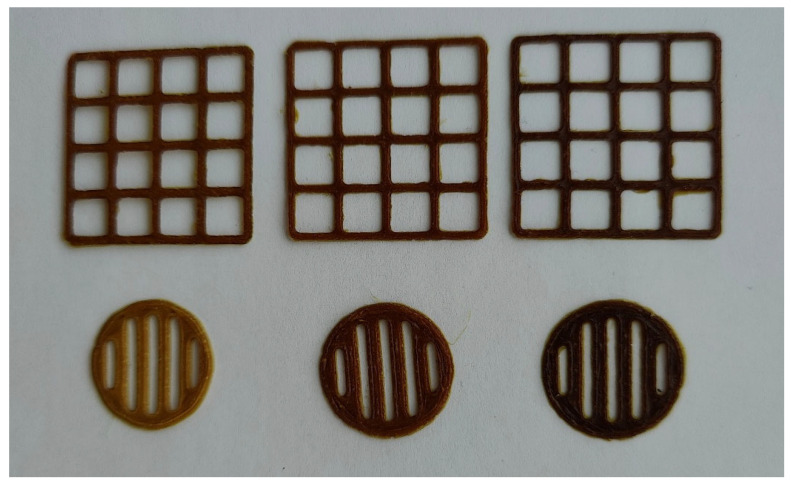
Photographs of the semi-solid extrusion (SSE) 3D-printed scaffolds loaded with *S. canadensis extract*. The content of extract in the printing gel (10 g) is 0.5, 1.0, and 1.5 g (from left to right).

The PEO printing gels loaded with dry *S. canadensis* extract at different concentrations appeared as viscous semisolids with a yellow-brown-greenish colour and a distinct odour. As seen in Figure 1, the gels were quite homogeneous in structure. The viscosity of gels was assessed at a rotational speed of 0.05 RPM and a shear rate of 0.100 1/s at a temperature of 22 ± 2 °C, and the results are summarised in Table 12. To investigate the feasibility of the present aqueous PEO gels loaded with *S. canadensis* extract for SSE 3D printing, standard square- and round-shaped scaffolds were printed (Figure 2). Table 12 summarises the average surface area, S_practical_/S_theoretical_ ratio, and mass (and mass variation) of the 3D-printed square-shaped lattice scaffolds and round-shaped discs.

## 4. Discussion

A total of 18 phenolic compounds were identified and quantified in the *S. canadensis* dry extract and its amino acid preparations. The most dominant compounds were hydroxycinnamic acids, such as neochlorogenic acid and chlorogenic acid, and additionally 4.5-dicaffeoylquinic acid, 3.5-dicaffeoylquinic acid, and 3.4-dicaffeoylquinic acid. Rutin and isoquercitrin were the primary flavonoids. Woźniak et al. (2018) reported that *S. canadensis* is also rich in flavonols (mainly quercetin and its glycosides) and has significant amounts of kaempferol derivatives [15]. Our findings are in accordance with the results reported in the literature showing the presence of quercetin compounds, but we found a notably lower content of kaempferol derivatives. Woźniak and co-workers (2018) also reported that caffeoylquinic acid esters form a major group of phenolic compounds. In this group, 5-*O*-caffeoylquinic acid (neochlorogenic acid) is the predominant one, accompanied by various mono- and di-caffeoylquinic acids and feruloylquinic acids [15]. In our extracts, however, the most abundant compounds were 3.4-dicaffeylquinic, 3.5-dicaffeylquinic, 4.5-dicaffeoylquinic acids, and chlorogenic acid, while ferulic acid derivatives were absent. According to the European Pharmacopoeia monograph for *Solidago herba*, flavonoids (more specifically hyperoside) are considered quality markers [14]. Nevertheless, our study revealed a predominance of rutin and a considerable presence of hydroxycinnamic acids. Therefore, in the standardisation of the dry extracts, these two categories of biologically active compounds should be considered. It is also important to note that the content of all phenolic compounds and their overall groups identified here was lower in the extracts modified with amino acids, which is associated with adding the amino acids. Modifying biologically active substances (BAS) through conjugation with amino acids is a well-established approach applied to isolated molecules and complex mixtures like fractions or plant extracts [26,28,29,30,31,32,33,34]. It is known that the conjugation of phenolic compounds with amino acids may affect their solubility, bioavailability, and transport to target sites, but this requires further in-depth molecular studies. The analytical methods we used were not capable of proving which chemical bond is between amino acids and phenolics, since during chromatography, the acidic medium breaks these bonds, and in the analysis, we detect the amino acid and the phenolic compound separately. To prove this, it is necessary to very carefully isolate these compounds individually and determine their structure. One thing is certain: some amino acids potentiate the pharmacological activity of plant extracts. Therefore, it would be interesting to further investigate how this affects their pharmacological activity.

A total of 14 amino acids were identified and quantified in the dry extract of *S. canadensis* and its amino acid preparations (including 7 essential ones). The predominant amino acids were proline, histidine, serine, alanine, aspartic acid, lysine, and glutamic acid. To our best knowledge, no research has been published to date providing such results on the amino acid composition of *S. canadensis* raw material or its extracts, thus making our findings novel and interesting.

**Figure 3:**
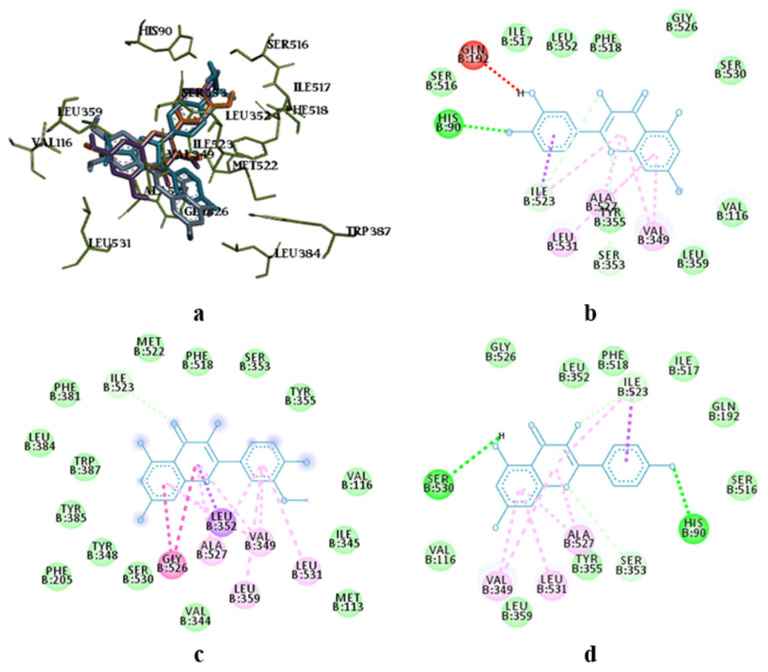
Superposition of flavonoids compared to celecoxib (**a**) and intermolecular interaction diagrams of quercetin (**b**), isorhamnetin (**c**), and kaempferol (**d**) in the active site of COX-1. The superposed molecules are shown in the following colours: celecoxib—blue, quercetin—orange, isorhamnetin—grey, and kaempferol—purple.

**Figure 4:**
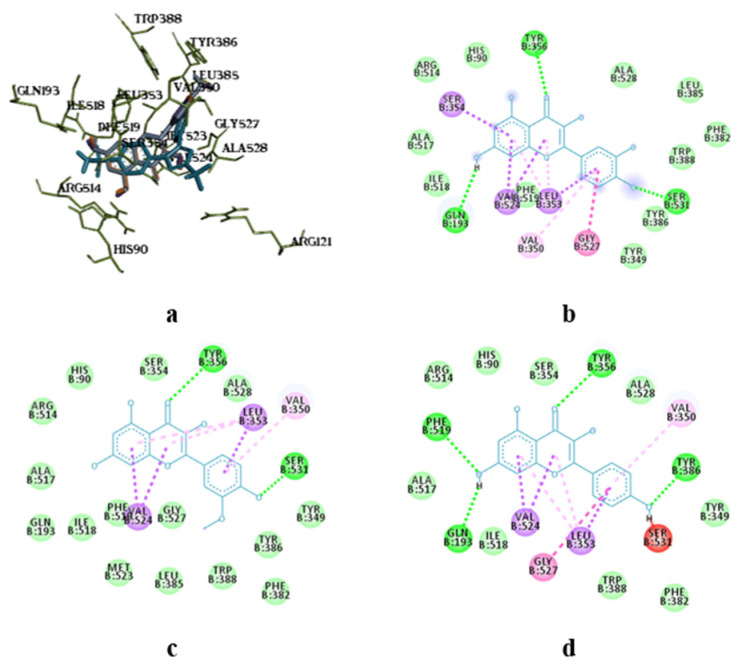
Superposition of flavonoids compared to celecoxib (**a**) and intermolecular interaction diagrams of quercetin (**b**), isorhamnetin (**c**), and kaempferol (**d**) in the active site of COX-2.

The molecular docking study showed the localisation of flavonoid fraction molecules relative to COX-1 and COX-2, revealing that the binding mode of such flavonoid molecules is similar to classical inhibitors. This is evidenced by the superposition of quercetin, isorhamnetin, and kaempferol within the docking site of celecoxib (Figure 3 and Figure 4).

In the case of quercetin and isorhamnetin complexes with COX-2, hydrogen bonds involving phenolic hydroxyl groups with the Ser531 residue play a crucial role in enzyme inhibition (Figure 4b,c). In the case of kaempferol, this interaction is negated due to forming an unfavourable bond with Ser531 (as seen in Figure 4d). Previously, it was shown that kaempferol has anti-BCRD effects by inhibiting the COX-2/PGE2 pathway, which regulates neuroinflammation [62]. Moreover, quercetin was shown to have better in-silico activity against COX-2 inhibition than diclofenac [63]. So, the findings of our current study confirm previous studies.

**Figure 5:**
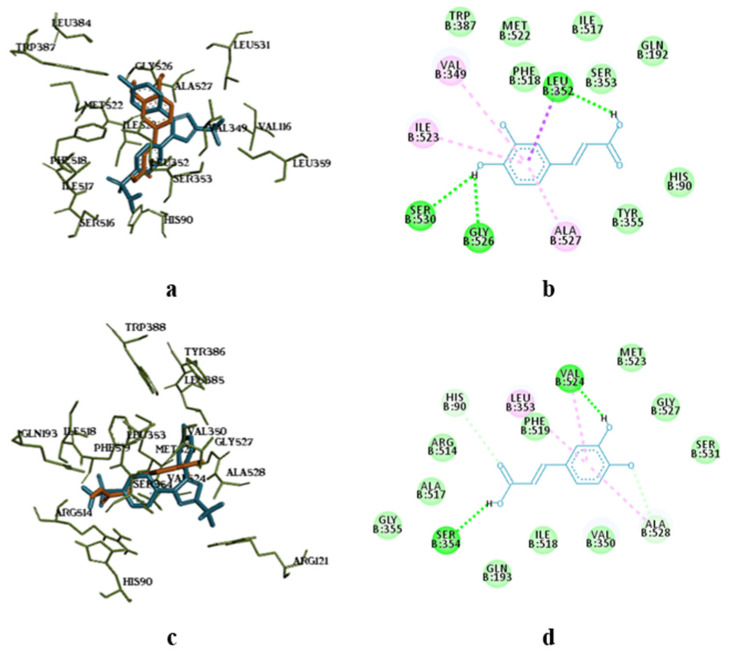
Superposition of caffeic acid compared to celecoxib (**a**,**c**) and intermolecular interaction diagrams (**b**,**d**) in the active sites of COX-1 and COX-2, respectively. The superposition of molecules is shown in the following colours: celecoxib—blue, caffeic acid—orange.

The molecular docking analysis of flavonoid complexes with the active site residues of cyclooxygenases revealed that the formation of a hydrophobic pocket (in addition to hydrogen bonds) contributes to the additional stability and strength of the complexes due to numerous hydrophobic interactions (π-σ, π-π, π-Alk). The binding of caffeic acid to the active sites of cyclooxygenases also occurs within the celecoxib-binding regions (as seen in Figure 5a,c). As shown in Figure 5b,d, caffeic acid forms a crucial hydrogen bond for activity expression through its phenolic hydroxyl group with the Ser530 residue and exhibits Van der Waals interactions with the Ser531 residue in the active sites of COX-1 and COX-2, respectively. Previously, caffeic acid was proven to inhibit COX-2 and was shown to be blocked peroxidase dependent. This converts prostaglandin G2 to prostaglandin H2, reduces superoxide production, and subsequently diminishes lipid peroxidation [64].

The *S. canadensis* extract and its amino acid preparations did not cause any fatalities when administered to mice at 5000 mg/kg body weight. The general condition of the animals remained satisfactory, and only a slight physiological body weight gain (increase) was observed. No alterations in biochemical parameters or morphological structure of internal organs were found in the test animals. Therefore, the findings of the acute toxicity study of the *S. canadensis* herb extracts confirm the absence of toxic effects after a single intragastric administration at a dose of 5000 mg/kg. This suggests that the median lethal dose (LD₅₀) exceeds the administered dose of 5000 mg/kg, and thus the present extracts can be classified as practically non-toxic preparations (toxicity class V, LD₅₀ > 5000 mg/kg).

As seen in Table 7, the *S. canadensis* extract and its amino acid preparations showed antimicrobial activity against *Staphylococcus aureus*, *Enterococcus faecalis,* and β-hemolytic *Streptococcus pyogenes*. The present dry extracts and preparations, however, did not inhibit the growth of microorganisms such as α-hemolytic *Streptococcus anginosus*, *Streptococcus pneumoniae*, *E. coli*, *E. coli* hly+, *Acinetobacter baumani*, *Pseudomonas aureginosa*, *Candida albicans*, *Candida lusitaniae*, and *Candida lipolytica*. Therefore, the present preparation had only a moderate antimicrobial activity and a relatively narrow microbiological spectrum. However, these studies are essential and important for the further development of standardisation methods, particularly when selecting a method for determining microbiological purity in accordance with the requirements of the European Pharmacopoeia [14].

As shown in Table 8 and Table 9, the inflammatory process in the rat paw in the control group was accompanied by an increase in its volume, which persisted until the end of the experiment. The administration of the extract preparations to the rats in groups 1–7 led to varying degrees of inhibition of the inflammatory response compared to the control group. The inhibition effect started in the first hour of the study. The most pronounced anti-inflammatory effect over the entire study period was found in the groups of animals that received the S, S-Phe, S-Arg, S-Gly, and S-Ala extracts at a dose of 100 mg/kg body weight. The total anti-inflammatory activity values were 53.89%, 47.84%, 51.87%, 49.03%, and 47.02%, respectively.

Within the first hour of the study, all extract preparations (except S-Phe) showed a higher inhibitory effect for the inflammatory reaction than that observed with quercetin. Within the first hour, the anti-inflammatory activity of two extracts (S-Arg and S-Val) was at the same level as shown with sodium diclofenac. The inflammatory activity of extract S (without any amino acids) exceeded 13.4%. After three hours, the most pronounced anti-inflammatory activity was observed with the S-Gly extract (5.7% higher than the corresponding activity shown with sodium diclofenac). In the animal group receiving S-Lys extract, the anti-inflammatory effect was close to that obtained with sodium diclofenac. The anti-inflammatory activity of the other extracts studied was close to the activity level of diclofenac or slightly lower. After five hours, the anti-inflammatory activity of four *S. canadensis* extracts (S, S-Phe, S-Arg, and S-Lys) increased, while the corresponding activity was slightly decreased with three extracts (S-Gly, S-Ala, and S-Val). After five hours, however, the anti-inflammatory activity of all extract preparations was higher than the anti-inflammatory activity of quercetin. Four *S. canadensis* extract preparations (S, S-Phe, S-Arg, and S-Gly) showed anti-inflammatory activity even higher than that found with sodium diclofenac by 21.98%, 24.60%, 16.16%, and 11.98%, respectively. The *S. canadensis* extracts studied here have anti-inflammatory activity in the formalin-induced oedema model. S and S-Arg *S. canadensis* extracts showed the most pronounced anti-inflammatory activity. According to our previous studies, aqueous and aqueous-ethanolic extracts of *S. canadensis* have anti-inflammatory activity [19,35], and the modification with amino acids potentiates this effect. Recently, Nkuimi Wandjou et al. [9] showed that *S. canadensis* essential oil has an anti-inflammatory effect. However, in our study, the concentration of *S. canadensis* essential oil is not high enough to show an anti-inflammatory effect as such due to the extractant used. Valverde et al. [6] isolated a labdane-type diterpenoid solidagenone from *S. chilensis*, and the authors reported that it significantly inhibited croton oil-, arachidonic acid- and phenol-induced ear oedema. Several studies have proven that phenolic compounds of *S. canadensis* have pronounced antioxidant activity [7,8,10], which directly correlates with anti-inflammatory activity. Therefore, considering the high content of phenolics in the extracts, such an anti-inflammatory effect is logical”.

As shown in Table 10, administering tetrachloromethane to the rodents (rats) of a control group led to a significant increase in LMI, thus indicating liver damage. With the rats receiving *S. canadensis* herb extract preparations or silymarin (a hepatoprotective drug), the changes in LMI were less pronounced compared to the intact animals of a control group. The highest hepatoprotective effect was observed with the *S. canadensis* extract preparation loaded with valine (S-Val), and the hepatoprotective activity was even higher than that induced with silymarin. The three extract preparations studied (S, S-Ala, and S-Lys) presented a hepatoprotective effect equal to silymarin, while S-Phe showed a slightly lower effect. The two extract preparations (S-Arg and S-Gly) showed a significantly lower hepatoprotective effect than the intact animals (Table 10). In summary, we showed that the *S. canadensis* herb extract preparations (and silymarin) have a hepatoprotective effect by reducing liver swelling and normalising organ circulation in rats, and consequently, decreasing the intensity of an inflammatory process.

Table 11 demonstrates that a single administration of tetrachloromethane in rats leads to the development of acute toxic liver damage. A significant enhancement of lipid peroxidation reactions was observed in the control group of rats. This could result in the depletion of an antioxidant defence system, the disruption of the structural and functional integrity of membranes, and the development of a pronounced cytolytic syndrome. The administration of *S. canadensis* extracts and silymarin caused a reduction in the levels of these biochemical indicators. The most pronounced effect was found with the *S. canadensis* extracts S and S-Phe, and the enzyme activity was decreased relative to the control group as follows: the ALT activity by 1.67 times (*p* < 0.05) and 2.09 times (*p* < 0.05), AST activity by 1.15 times (*p* < 0.05) and 1.42 times (*p* < 0.05), and ALP activity by 1.62 times (*p* < 0.05) and 1.74 times (*p* < 0.05), respectively. The S. canadensis extract preparations S-Ala and S-Lys presented a slightly lower effect on the development of cytolysis syndrome. These two extract preparations (S-Ala and S-Lys) reduced the ALT activity by 1.21 times (*p* < 0.05) and 1.37 times (*p* < 0.05), AST activity by 1.09 times and 1.30 times (*p* < 0.05), and ALP activity by 1.46 times (*p* < 0.05) and 1.56 times (*p* < 0.05), respectively.

The administration of *S. canadensis* extract preparations S-Arg, S-Gly, and S-Val (and silymarin) to rats resulted in a slight decrease in the ALT activity by 6.7%, 7.0%, and 11.8% (8.0%) (*p* < 0.05), AST activity by 1.0%, 2.1%, and 6.8% (4.2%), and ALP activity by 11.1%, 8.0%, and 19.4% (*p* < 0.05) (15.6%, *p* < 0.05) compared to the control group. The administration of the *S. canadensis* extract (S) to rats decreased the enzyme activity compared to the administration of silymarin: the ALT activity increased by 1.54 times (*p* < 0.05), AST activity by 1.11 times, and ALP activity by 1.13 times (*p* < 0.05). The inclusion of phenylalanine (S-Phe), alanine (S-Ala), and lysine (S-Lys) in *S. canadensis* extract also enhanced the hepatoprotective activity of the extracts. After the administration of the extract preparations S-Phe, S-Ala, and S-Lys, the ALT activity in rats decreased by 1.92 times (*p* < 0.05), 1.11 times, and 1.26 times (*p* < 0.05); AST activity by 1.36 times (*p* < 0.05), 1.04 times, and 1.24 times (*p* < 0.05); and ALP activity by 1.49 times, 1.24 times, and 1.32 times (*p* < 0.05) compared to the silymarin group (Table 11).

The concomitant administration of a hepatotropic toxin and *S. canadensis* extracts (S, S-Phe, S-Ala, S-Lys, S-Val) at a dose of 25 mg/kg body weight resulted in a significant reduction in the TBK-AP level in the liver homogenate of rats compared to the control animal group. The TBK-AP levels found were 1.74 times (S), 2.15 times (S-Phe), 1.93 times (S-Ala), 2.14 (S-Lys), and 1.51 times (*p* < 0.05) lower than the TBK-AP levels observed with the control animal group. The administration of the other extract preparations, S4, S5, and S9, did not change the TBK-AP level in the liver tissues of rats compared to the control group. The use of silymarin resulted in a 1.36-fold (*p* < 0.05) decrease in the level of TBK-reactants in the liver homogenate of rats compared to the control group.

The *S. canadensis* extract (S) and the extract preparations loaded with phenylalanine (S-Phe), alanine (S-Ala), and lysine (S-Lys) reduced the TBK-AP level (compared to a silymarin group) by 1.27, 1.58, 1.42, and 1.56 times, respectively (*p* < 0.05). The hepatoprotective activity of S-Val was equal to the activity found with silymarin, while the use of extracts S-Arg and S-Gly did not improve the levels of antioxidant system indicators compared to the use of silymarin. In summary, the results suggest that *S. canadensis* extracts present a clear hepatoprotective activity by inhibiting peroxide destructive processes and reducing the development of cytolysis syndrome under the acute toxic hepatitis induced by tetrachloromethane. The *S. canadensis* extract preparations S, S-Phe, S-Ala, and S-Lys showed an even higher hepatoprotective effect than a hepatoprotective drug, silymarin. According to the literature, aqueous and aqueous-ethanolic extracts of *S. canadensis* have hepatoprotective activity [19,35], and the modification with amino acids potentiates this effect.

The aqueous PEO printing gels loaded with 0.5–1.5 g of *S. canadensis* extract (in 10 mL of gel) were feasible for SSE 3D printing. The 3D-printed lattices and round-shaped discs were uniform in shape and size (Figure 2). Our results also confirm the compatibility of PEO (career polymer) with *S. canadensis* extract in gel inks. In our previous studies, the 3D-printed preparations for German chamomile [26] and eucalyptus extracts [58] were designed and developed using pre-selected surface-active agents in the printing formulations.

The results of the pilot disintegration test in vitro showed that the SSE 3D-printed PEO round-shaped discs with *S. canadensis* extract completely lost their shape and disintegrated within 15–20 min in purified water at room temperature at 22 ± 2 °C. We also conducted a complementary disintegration test according to the European Pharmacopoeia (Ph.Eur.) [14], and the results confirmed the results obtained in a pilot disintegration test in vitro. The results of the *in-vitro* disintegration test (Ph.Eur.) showed that all 3D-printed preparations tested were completely deformed within 10 min and fully disintegrated within 20–27 min. The final disintegration time was dependent on the *S. canadensis* extract concentration (the higher concentration retarded the disintegration). However, since all 3D-printed preparations disintegrated within 30 min, this suggests their potential as immediate-release delivery systems for oral administration [65,66].

After the development of standardisation methods for the present 3D-printed herbal dosage forms, it would also be important to conduct preclinical and clinical studies to confirm their safety and efficacy. Moreover, all pharmaceutical development stages need to be successfully completed to verify the pharmaceutical, chemical, and microbiological quality of the preparations. Today, regulatory requirements and marketing authorisation procedures for 3D-printed medicinal products are not very clear and established. This is an additional challenge for the present new 3D-printed herbal preparations of *S. canadensis* extract and could hinder adopting the use of such preparations for a wider population.

## 5. Conclusions

The *S. canadensis* dry extract studied here consists of 18 phenolic compounds and 14 amino acids. The present dry extract and its amino acid-modified formulations have anti-inflammatory and hepatoprotective activity verified with rodent models. The dry extract also has moderate antimicrobial activity against *Staphylococcus aureus*, *Enterococcus faecalis,* and β-hemolytic *Streptococcus pyogenes*. Based on the molecular docking results, the flavonoid fraction and caffeic acid in the *S. canadensis* extracts exhibit moderate inhibitory activity on the binding modes in the active sites of COX-1 and COX-2. The results of the acute toxicity study with mice confirm the absence of toxic effects of *S. canadensis* herb extract and its amino acid preparations after the single intragastric administration of such preparations at 5000 mg/kg body weight. Consequently, *S. canadensis* dry extract and its amino acid-modified extracts can be classified as practically non-toxic preparations (toxicity class V, LD₅₀ > 5000 mg/kg). The most pronounced hepatoprotective activity was found with *S. canadensis* herb extract and its amino acid preparations combined with phenylalanine, alanine, and lysine at a dose of 25 mg/kg body weight. These extracts significantly outperformed the reference drug (silymarin) in terms of liver mass coefficient, enzyme activities (ALT, AST, and ALP), and the levels of lipid peroxidation products (TBA-RS). Moreover, the biochemical parameters of blood and liver homogenate found in the treated animals were close to the corresponding parameters found in the animals in a control group. The results suggest that the most pronounced anti-inflammatory activity can be obtained with the *S. canadensis* herb extract and its preparation with arginine. The development of innovative 3D-printed oral dosage forms represents a promising strategy for these herbal extracts in gaining their applications as dietary supplements or pharmaceutical products. However, further research is needed to complete standardisation and preclinical and clinical studies of the present novel 3D-printed oral dosage form of *S. canadensis* herb extract.

## Figures and Tables

**Table 1:** Content of phenolics in the *S. canadensis* extract and its amino acid preparations.

Compound	Content in the Dry Extract, mg/g						
	S [19]	S-Phe	S-Arg	S-Gly	S-β-Ala	S-Lys	S-Val
Neochlorogenic acid	0.86 ± 0.08	0.78 ± 0.01	0.78 ± 0.02	0.94 ± 0.02	0.87 ± 0.01	0.85 ± 0.03	0.84 ± 0.02
Kaempferol-3-*O*-rutinoside	0.21 ± 0.08	0.20 ± 0.01	0,21 ± 0.02	0.20 ± 0.02	0.20 ± 0.01	0.20 ± 0.03	0.22 ± 0.03
Isoquercitrin	8.07 ± 0.24	0.40 ± 0.03	0,42 ± 0.02	0.45 ± 0.01	0.46 ± 0.02	0.46 ± 0.02	0.43 ± 0,034
Chlorogenic acid	11.87 ± 0.42	14.34 ± 0.44	14.53 ± 0.17	16.54 ± 0.34	15.57 ± 1.42	15.62 ± 0.23	15.40 ± 0.41
Quercetin	8.43 ± 0.19	5.964 ± 0.29	6.83 ± 0.19	6.87 ± 0.08	6.50 ± 0.27	8.21 ± 0.22	6.85 ± 0.10
Isorhamnetin-3-*O*-rutinoside	2.98 ± 0.21	0.31 ± 0.03	0.32 ± 0.00	0.36 ± 0.01	0.31 ± 0.02	0.35 ± 0.01	0.36 ± 0.03
*p*-Coumaric acid	0.05 ± 0.01	0.04 ± 0,01	0.04 ± 0.00	0.05 ± 0.01	0.04 ± 0.01	0.04 ± 0.01	0.04 ± 0.01
Ferulic acid	0.05 ± 0.01	0.05 ± 0.01	0,04 ± 0.00	0.05 ± 0.00	0.05 ± 0.00	0.04 ± 0.01	0.04 ± 0.01
Vanilic acid	0.21 ± 0.03	0.16 ± 0.01	0.16 ± 0.01	0.17 ± 0.01	0.17 ± 0.01	0.15 ± 0.01	0.15 ± 0.02
Caffeic acid	0.19 ± 0.01	0.22 ± 0.02	0.24 ± 0.01	0.26 ± 002	0.22 ± 0.01	0.27 ± 0.01	0.23 ± 0.01
Kaempferol	2.67 ± 0.35	2.14 ± 0.08	2.28 ± 0.04	2.27 ± 0.15	2.13 ± 0.18	2.71 ± 0.30	2.45 ± 0.11
3,4-Dihydroxy-phenylacetic acid	1.71 ± 0.11	0.58 ± 0.01	0.62 ± 0.01	0.85 ± 0.02	0.71 ± 0.02	0.64 ± 0.03	0.60 ± 0.02
Isorhamnetin	1.08 ± 0.18	0.74 ± 0.01	0.80 ± 0.01	0.80 ± 0.01	0.82 ± 0.03	0.99 ± 0.02	0.81 ± 0.01
Rutin	28.23 ± 0.42	4.76 ± 0.06	5.02 ± 0.22	5.35 ± 0.10	4.85 ± 0.26	5.55 ± 0.20	5.33 ± 0.15
Hyperoside	0.42 ± 0.05	0.12 ± 0.00	0.13 ± 0.01	0.13 ± 0.01	0.12 ± 0.02	0.15 ± 0.01	0.13 ± 0.00
4.5-Dicaffeoylquinic acid	3.06 ± 0.31	1.82 ± 0.14	1.88 ± 0.12	2.20 ± 0.22	2.33 ± 0.14	2.29 ± 0.17	2.26 ± 0.07
3.5-Dicaffeylquinic acid	4.86 ± 0.27	3.67 ± 0.05	3.99 ± 0.42	4.24 ± 0.29	3.93 ± 0.14	4.71 ± 0.18	4.74 ± 0.18
3.4-Dicaffeylquinic acid	25.42 ± 0.53	21.25 ± 0.04	24.23 ± 2.14	25.44 ± 2.39	21.78 ± 2.38	28.11 ±1.15	28.45 ± 2.53
Hydroxycinnamic acids (chlorogenic acid equivalents, spectrophotometry), %	5.34 ± 0.42	4.93 ± 0.36	2.73 ± 0.26	4.34 ± 0.17	5.07 ± 0.32	3.22 ± 0.29	4.42 ± 0.21
Flavonoids (rutin equivalents, spectrophotometry), %	9.68 ± 0.14	5.18 ± 0.36	6.07 ± 0.47	6.51 ± 0.13	7.20 ± 0.06	7.55 ± 0.11	6.72 ± 0.15
Total phenolic compounds (gallic acid equivalents, spectrophotometry), %	11.56 ± 0.28	7.61 ± 0.22	7.19 ± 0.50	7.28 ± 0.09	7.68 ± 0.24	7.34 ± 0.19	7.88 ± 0.37

Notes: S-the dry *S. canadensis* extract, obtained with 40% aqueous ethanol, and its amino acid preparations with phenylalanine (S-Phe), arginine (S-Arg), glycine (S-Gly), β-alanine (S-β-Ala), lysine (S-Lys), and valine (S-Val).

**Table 2:** Amino acid composition of the *S. canadensis* extract and its amino acid preparations.

Compound	Content in the Dry Extract, mg/g						
	S [19]	S-Phe	S-Arg	S-Gly	S-β-Ala	S-Lys	S-Val
Alanine	2.09 ± 0.07	1.71 ± 0.13	1.61 ± 0.06	1.95 ± 0.09	1.89 ± 0.32	1.63 ± 0.19	2.02 ± 0.15
Arginine	1.72 ± 0.05	4.45 ± 0.31	91.67 ± 3.78	8.01 ± 0.37	2.23 ± 0.24	4.23 ± 0.24	2.03 ± 0.12
Aspartic acid	2.26 ± 0.08	2.05 ± 0.20	2.02 ± 0.22	2.16 ± 0.13	1.59 ± 0.22	1.85 ± 0.07	2.14 ± 0.22
Glutamic acid	2.01 ± 0.11	1.75 ± 0.05	1.65 ± 0.14	1.78 ± 0.07	1.56 ± 0.10	1.60 ± 0.02	1.74 ± 0.02
Glycine	0.32 ± 0.04	0.32 ± 0.01	0.99 ± 0.08	92.67 ± 6.40	0.51 ± 0.05	0.28 ± 0.07	0.38 ± 0.07
Histidine	1.12 ± 0.03	0.88 ± 0.04	0.05 ± 0.00	0.76 ± 0.11	1.10 ± 0.01	1.26 ± 0.07	1.21 ± 0.09
Isoleucine	0.87 ± 0.04	1.08 ± 0.05	0.45 ± 0.03	0.60 ± 0.18	0.67 ± 0.08	0.25 ± 0.05	0.67 ± 0.08
Leucine	0.79 ± 0.02	1.24 ± 0.12	0.71 ± 0.02	0.83 ± 0.06	0.75 ± 0.07	0.67 ± 0.03	2.18 ± 0.14
Lysine	1.31 ± 0.05	0.98 ± 0.12	0.84 ± 0.10	6.78 ± 0.33	2.29 ± 0.13	231.10 ± 11.61	3.60 ± 0.11
Phenylalanine	1.54 ± 0.06	135.51 ± 10.47	0.65 ± 0.02	0.55 ± 0.01	0.49 ± 0.03	0.41 ± 0.02	0.56 ± 0.06
Proline	7.32 ± 0.07	6.60 ± 0.14	6.79 ± 0.27	7.80 ± 0.23	7.00 ± 0.10	6.88 ± 0.13	9.16 ± 0.09
Serine	1.76 ± 0.02	1.65 ± 0.10	1.50 ± 0.04	1.81 ± 0.10	1.46 ± 0.09	1.48 ± 0.05	1.60 ± 0.12
Threonine	0.75 ± 0.03	0.64 ± 0.09	0.54 ± 0.16	0.78 ± 0.43	0.51 ± 0.21	0.88 ± 0.05	0.63 ± 0.27
Valine	0.95 ± 0.04	1.60 ± 0.20	0.49 ± 0.05	0.60 ± 0.02	0.55 ± 0.04	0.99 ± 0.04	85.87 ± 3.81
β-Alanine	-	-	-	-	127.63 ± 4.75		

**Table 3:** The molecular docking values for the molecules in the COX-1 and COX-2 binding sites.

Molecules	COX-1			COX-2		
	Affinity DG ^1^	EDoc ^2^	Ki ^3^	Affinity DG ^1^	EDoc ^2^	Ki ^3^
Quercetin	−8.8	−4.81	297.39 μM	−8.8	−5.63	75.03 μM
Isorhamnetin	−8.6	−5.11	179.54 μM	−8.9	−4.48	516.88 μM
Kaempferol	−9.2	−5.28	134.94 μM	−8.7	−6.07	35.27 μM
Chlorogenic acid	−7.5	−1.85	43.74 mM	−7.1	−2.09	29.57 mM
Neochlorogenic acid	−8.6	−3.57	2.42 mM	−7.4	−4.10	994.77 μM
4,5-Dicaffeoylquinic acid	−7.7	+7.51	-	−7.3	−0.99	188.10 mM
3,5-Dicaffeoylquinic acid	−9.0	+2.68	-	−6.4	−0.67	323.17 mM
3,4-Dicaffeoylquinic acid	−9.0	+0.98	-	−7.1	−0.96	198.68 mM
Caffeic acid	−7.5	−4.78	314.41 μM	−7.1	−3.53	2.60 mM
Quinic acid	−6.1	−1.65	61.23 mM	−6.1	−1.40	93.74 mM
Celecoxib	−10.9	−8.65	453.15 nM	−11.9	−9.87	58.02 nM

Notes: ^1^ Scoring function, kcal/mol; ^2^ Binding free energy, kcal/mol; ^3^ Binding constant (mM—millimolar, μM—micromolar, nM—nanomolar).

**Table 4:** Change in the body weight of mice after a single administration of *S. canadensis* extracts.

Group of Animals	$\mathrm{Body}\;\mathrm{Weight}\;\mathrm{of}\;\mathrm{White}\;\mathrm{Mice},\;g,\;\bar{x}\pm \Delta \bar{x}$, n = 6			
	Before the Experiment Begins	3 Days	7 Days	14 Days
1 (Extract S)	21.95 ± 0.75	22.50 ± 0.71	23.15 ± 0.88 *	23.83 ± 0.85 *
2 (Extract S-Phe)	23.40 ± 0.88	23.82 ± 0.86	24.43 ± 0.91	25.08 ± 0.82 *
3 (Extract S-Arg)	20.32 ± 1.06	20.87 ± 1.14	21.28 ± 1.05	21.90 ± 1.13 *
4 (Extract S-Gly)	23.02 ± 0.96	23.33 ± 1.00	23.88 ± 1.01	24.53 ± 1.06 *
5 (Extract S-Ala)	20.87 ± 0.96	21.28 ± 0.89	21.82 ± 0.95	22.50 ± 0.80 *
6 (Extract S-Lys)	23.20 ± 1.17	23.62 ± 1.12	24.22 ± 1.15	24.85 ± 1.13 *
7 (Extract S-Val)	22.27 ± 0.61	22.70 ± 0.55	23.27 ± 0.54 *	23.88 ± 0.49 *
Intact animals (water purified)	18.97 ± 0.55	19.50 ± 0.57	19.88 ± 0.49 *	20.52 ± 0.39 *

Note: * deviation of the indicator is significant with respect to the initial animal data (*p* < 0.05).

**Table 5:** Mass of internal organs in mice after a single administration of *Solidago canadensis* herb extracts.

Group of Animals	$\mathrm{Animal}\;\mathrm{Organ}\;\mathrm{Mass},\;g;\;\bar{x}\pm \Delta \bar{x}$, n = 6		
	Liver	Heart	Kidneys
1 (Extract S)	1.31 ± 0.032	0.11 ± 0.009	0.28 ± 0.020
2 (Extract S-Phe)	1.33 ± 0.069	0.12 ± 0.007	0.32 ± 0.017
3 (Extract S-Arg)	1.30 ± 0.039	0.09 ± 0.004	0.29 ± 0.015
4 (Extract S-Gly)	1.28 ± 0.054	0.10 ± 0.009	0.28 ± 0.031
5 (Extract S-Ala)	1.26 ± 0.028	0.10 ± 0.005	0.27 ± 0.020
6 (Extract S-Lys)	1.32 ± 0.034	0.10 ± 0.008	0.31 ± 0.025
7 (Extract S-Val)	1.28 ± 0.031	0.11 ± 0.013	0.28 ± 0.020
Intact animals (water purified)	1.25 ± 0.020	0.09 ± 0.012	0.27 ± 0.025

**Table 6:** Biochemical parameters of blood in mice after 14 days of a single administration of modified extracts of *S. canadensis* (goldenrod) herb.

Group of Animals	$\mathrm{Indicator},\;\bar{x}\pm \Delta \bar{x}$, n = 6		
	ALT, µmol/h·mL	AST, µmol/h·mL	de Ritis Ratio
1 (Extract S)	0.26 ± 0.031	0.30 ± 0.035	1.15
2 (Extract S-Phe)	0.29 ± 0.029	0.32 ± 0.046	1.10
3 (Extract S-Arg)	0.26 ± 0.034	0.30 ± 0.042	1.20
4 (Extract S-Gly)	0.27 ± 0.033	0.34 ± 0.039	1.26
5 (Extract S-Ala)	0.25 ± 0.043	0.31 ± 0.024	1.24
6 (Extract S-Lys)	0.30 ± 0.054	0.32 ± 0.038	1.07
7 (Extract S-Val)	0.28 ± 0.039	0.31 ± 0.040	1.11
Intact animals (purified water)	0.29 ± 0.023	0.31 ± 0.033	1.09

**Table 7:** Antimicrobial activity and antifungal activity of *S. canadensis* extracts at a concentration of 100 mg/mL (diameters of growth inhibition zones, mm).

Microorganisms			Control Ethanol 40%	S	S-Phe	S-Arg	S-Gly	S-Ala	S-Lys	S-Val
Species	Clinical Material	Resistance								
*Staphylococcus aureus*	Pharynx	BSSA	growth	13.68 ± 0.39	11.31 ± 0.50	growth	10.34 ± 1.00	13.77 ± 0.32	10.58 ± 0.19	11.78 ± 0.74
*Staphylococcus aureus*	Wound	BSSA, MLs	growth	13.09 ± 0.64	11.44 ± 0.28	growth	10.51 ± 0.28	13.37 ± 0.50	11.38 ± 1.46	11.62 ± 0.74
*Enterococcus faecalis*	Urethra	Tet, FQin	growth	growth	growth	13.79 ± 0.49	12.55 ± 1.11	12.09 ± 0.56	0	11.86 ± 0.71
*β-hemolytic Streptococcus pyogenes*	Pharynx	S	14.60 ± 2.28	growth	growth	18.18 ± 0.69	growth	growth	growth	growth
*α-hemolytic Streptococcus anginosus*	Pharynx	AMO, Tet, MLs	15.51 ± 1.28	growth	growth	growth	growth	growth	growth	growth
*Streptococcus pneumoniae*	Sputum	S	growth	growth	growth	growth	growth	growth	growth	growth
*Streptococcus pneumoniae*	Sputum	b-Lac, Tet	growth	growth	growth	growth	growth	growth	growth	growth
*E. coli*	Wound	S	growth	growth	growth	growth	growth	growth	growth	growth
*E. coli*	Wound	S	growth	growth	growth	growth	growth	growth	growth	growth
*E. coli*	Wound	AMO Tet, FQin	growth	growth	growth	growth	growth	growth	growth	growth
*E. coli hly+*	Faeces	AMO, MLs	growth	growth	growth	growth	growth	growth	growth	growth
*Acinetobacter baumani*	Sputum	ESbL	growth	growth	growth	growth	growth	growth	growth	growth
*Pseudomonas aureginosa*	Wound	ESbL	growth	growth	growth	growth	growth	growth	growth	growth
*Candida albicans*	Oral cavity	FCZ-R	growth	growth	growth	growth	growth	growth	growth	growth
*Candida albicans*	Sputum	FCZ-R	10.54 ± 0.62	growth	growth	growth	growth	growth	growth	growth
*Candida albicans*	Urine	FCZ-R	growth	growth	growth	growth	growth	growth	growth	growth
*Candida albicans*	Oral cavity	FCZ-S	growth	growth	growth	growth	growth	growth	growth	growth
*Candida lusitaniae*	Oral cavity	FCZ-R	growth	growth	growth	growth	growth	growth	growth	growth
*Candida lipolytica*	Oral cavity	FCZ-S	growth	growth	growth	growth	growth	growth	growth	growth

**Table 8:** Effect of *S. canadensis* extracts on the development of limb oedema in rats.

Group of Animals	Dose, mg/100 g	$\mathrm{Rat}\;\mathrm{Paw}\;\mathrm{Volume}\;\mathrm{Increase},\;\%:\;\bar{x}\pm \Delta \bar{x}$, n = 7		
		in 1 h	in 1 h	in 5 h
1 (Extract S)	10	18.33 ± 4.21 */#	22.89 ± 5.23 *	16.57 ± 4.46 */#
2 (Extract S-Phe)	10	25.51 ± 6.80	22.06 ± 4.12 *	15.93 ± 3.73 */#
3 (Extract S-Arg)	10	19.86 ± 3.07 *	22.17 ± 5.17 *	18.00 ± 2.89 */#
4 (Extract S-Gly)	10	24.75 ± 6.84 *	18.30 ± 4.25 */#	19.05 ± 4.17 */#
5 (Extract S-Ala)	10	22.79 ± 6.21 *	21.29 ± 6.09 *	21.60 ± 5.91 */#
6 (Extract S-Lys)	10	21.37 ± 4.79 *	29.30 ± 5.19 *	25.78 ± 6.51 *
7 (Extract S-Val)	10	20.40 ± 5.48 *	24.15 ± 7.10 *	27.10 ± 6.69 *
8 (Sodium diclofenac)	0.8	20.17 ± 3.18 *	19.86 ± 3.08 *	21.97 ± 4.13 *
9 (Quercetin)	0.5	24.22 ± 4.55	28.49 ± 6.06 *	32.43 ± 5.76 */**
10 (Control group)	-	33.74 ± 6.73	47.33 ± 10.68	46.52 ± 11.45

Note: * probability of deviations from the data of the control group (*p* < 0.05); ** probability of deviations from the data of the group of animals that received diclofenac sodium (*p* < 0.05); # probability of deviation from the data of the group of animals that received quercetin (*p* < 0.05).

**Table 9:** Anti-inflammatory activity of the *S. canadensis* extracts in rats.

Group of Animals	Inflammatory Response Suppression Index, %			Total Anti-Inflammatory Activity, %
	in 1 h	in 1 h	in 5 h	
1 (Extract S)	45.67	51.64	64.37	53.89
2 (Extract S-Phe)	24.37	53.41	65.75	47.84
3 (Extract S-Arg)	41.15	53.17	61.30	51.87
4 (Extract S-Gly)	26.65	61.34	59.09	49.03
5 (Extract S-Ala)	32.46	55.03	53.56	47.02
6 (Extract S-Lys)	36.65	38.10	44.58	39.78
7 (Extract S-Val)	39.54	48.98	41.75	43.42
8 (Sodium diclofenac)	40.26	58.05	52.77	50.36
9 (Quercetin)	28.22	39.81	30.28	32.77

**Table 10:** Coefficient of liver mass of experimental animals ($\bar{x}\pm \Delta \bar{x}$, n = 7).

Group of Animals	m_animal_, g	m_liver_, g	LMI, %
1 (Intact animals)	145.00 ± 11.64	4.59 ± 0.56	3.16 ± 0.26
2 (Control group, CCl4)	153.29 ± 13.79	8.40 ± 1.19	5.45 ± 0.36 *
3 (Extract S)	188.29 ± 15.49	6.57 ± 0.89	3.63 ± 0.2 */**
4 (Extract S-Phe)	203.29 ± 19.74	7.79 ± 0.78	3.83 ± 0.14 */**/#
5 (Extract S-Arg)	186.57 ± 25.98	7.78 ± 1.80	4.15 ± 0.48 */**/#
6 (Extract S-Gly)	181.57 ± 15.49	8.09 ± 1.38	4.51 ± 0.98 */**/#
7 (Extract S-Ala)	220.00 ± 16.02	8.02 ± 0.60	3.60 ± 0.11 */**
8 (Extract S-Lys)	213.9 ± 23.68	7.64 ± 0.85	3.61 ± 0.44 **
9 (Extract S-Val)	216.57 ± 11.54	7.26 ± 1.03	3.34 ± 0.37 **
10 (Silymarin)	220.00 ± 11.94	7.93 ± 0.82	3.60 ± 0.22 */**

Notes: * probability of deviation from the data of the intact animal group (*p* < 0.05); ** probability of deviation from the data of the control animal group (*p* < 0.05); # probability of deviation from the data of the group of animals receiving silymarin (*p* < 0.05).

**Table 11:** The effect of *S. canadensis* extracts on the course of acute toxic hepatitis in rats caused by the administration of carbon tetrachloride (M ± m).

Group of Animals	Biochemical Indicators			
	Blood Serum			Liver Homogenate
	ALT, μmol/h·mL	AST, μmol/h·mL	ALP, nmol/s·L	TBK-AP nmol/g
1 (Intact animals)	1.55 ± 0.13	2.23 ± 0.28	1859.14 ± 177.67	18.63 ± 2.68
2 (Control group, CCl4)	4.64 ± 0.31 *	3.83 ± 0.24 *	4913.29 ± 465.37 *	51.60 ± 8.58 *
3 (Extract S)	2.78 ± 0.39 */**/#	3.32 ± 0.47 */**	3025.29 ± 442.29 */**/#	29.72 ± 3.40 */**/#
4 (Extract S-Phe)	2.22 ± 0.36 */**/#	2.69 ± 0.44 */**/#	2783.86 ± 332.95 */**/#	23.98 ± 5.69 **/#
5 (Extract S-Arg)	4.33 ± 0.50 *	3.82 ± 0.25 *	4366.86 ± 483.42 *	47.40 ± 10.35 *
6 (Extract S-Gly)	4.30 ± 0.42 *	3.75 ± 0.61 *	4520.00 ± 741.14 *	48.81 ± 5.86 */#
7 (Extract S-Ala)	3.84 ± 0.54 */**	3.52 ± 0.51 *	3354.86 ± 407.24 */**/#	26.71 ± 7.69 */**/#
8 (Extract S-Lys)	3.39 ± 0.56 */**/#	2.95 ± 0.46 */**/#	3148.29 ± 451.68 */**/#	24.10 ± 4.44 */**/#
9 (Extract S-Val)	4.09 ± 0.40 */**	3.57 ± 0.37 *	3962.00 ± 435.33 */**	34.22 ± 6.20 */**
10 (Silymarin)	4.27 ± 0.39 *	3.67 ± 0.37 *	4146.71 ± 610.78 */**	37.82 ± 3.71 */**

Notes: * probability of deviation from the data of the intact animal group (*p* < 0.05); ** probability of deviation from the data of the control animal group (*p* < 0.05); # probability of deviation from the data of the group of animals receiving silymarin (*p* < 0.05).

**Table 12:** Physical properties of the polyethylene oxide (PEO) printing gels loaded with *S. canadensis* extract and the corresponding 3D-printed scaffolds.

The Amount of Extract (g) in the Printing Gel (10 g)	Viscosity, cP (22 ± 2 °C)	Surface Area of the 3D-Printed Lattices, mm^2^	Spractical/ Stheoretical	Mass of Lattices, mg	Mass of Round-Shaped Discs, mg
0.5	126,867 ± 4958	347.72 ± 19.56	1.07	176.4 ± 2.1	125.7 ± 1.1
1.0	125,233 ± 5132	354.36 ± 27.97	1.09	206.7 ± 2.7	148.0 ± 4.6
1.5	102,967 ± 1775	373.13 ± 29.34	1.15	220.3 ± 2.5	154.4 ± 0.6

## Data Availability

The data supporting the results of this study can be obtained from the corresponding authors upon reasonable request.

## References

[1] Pal R.W., Chen S., Nagy D.U., Callaway R.M. (2015). Impacts of Solidago Gigantea on Other Species at Home and Away. Biol. Invasions.

[2] Poljuha D., Sladonja B., Uzelac Božac M., Šola I., Damijanić D., Weber T. (2024). The Invasive Alien Plant *Solidago canadensis*: Phytochemical Composition, Ecosystem Service Potential, and Application in Bioeconomy. Plants.

[3] Elshafie H.S., Gruľová D., Baranová B., Caputo L., De Martino L., Sedlák V., Camele I., De Feo V. (2019). Antimicrobial Activity and Chemical Composition of Essential Oil Extracted from *Solidago canadensis* L. Growing Wild in Slovakia. Molecules.

[4] Shelepova O., Vinogradova Y., Vergun O., Grygorieva O., Brindza J. (2020). Assessment of Flavonoids and Phenolic Compound Accumulation in Invasive *Solidago canadensis* L. in Slovakia. Potravin. Slovak J. Food Sci..

[5] Shelepova O., Vinogradova Y., Zaitchik B., Ruzhitsky A., Grygorieva O., Brindza J. (2018). Constituents of the Essential Oil in *Solidago canadensis* L. from Eurasia. Potravin. Slovak J. Food Sci..

[6] Valverde S.S., Santos B.C.S., De Oliveira T.B., Gonçalves G.C., De Sousa O.V. (2021). Solidagenone from *Solidago chilensis* Meyen Inhibits Skin Inflammation in Experimental Models. Basic Clin. Pharmacol. Toxicol..

[7] Deng Y., Zhao Y., Padilla-Zakour O., Yang G. (2015). Polyphenols, Antioxidant and Antimicrobial Activities of Leaf and Bark Extracts of *Solidago canadensis* L. Ind. Crops Prod..

[8] Apáti P., Szentmihályi K., Kristó S.T., Papp I., Vinkler P., Szoke É., Kéry Á. (2003). Herbal Remedies of Solidago—Correlation of Phytochemical Characteristics and Antioxidative Properties. J. Pharm. Biomed. Anal..

[9] Nkuimi Wandjou J.G., Quassinti L., Gudžinskas Z., Nagy D.U., Cianfaglione K., Bramucci M., Maggi F. (2020). Chemical Composition and Antiproliferative Effect of Essential Oils of Four *Solidago* Species (*S. canadensis*, *S. gigantea*, *S. virgaurea* and *S*.×*Niederederi*). Chem. Biodivers..

[10] Kraujalienė V., Pukalskas A., Venskutonis P.R. (2017). Biorefining of Goldenrod (*Solidago virgaurea* L.) Leaves by Supercritical Fluid and Pressurized Liquid Extraction and Evaluation of Antioxidant Properties and Main Phytochemicals in the Fractions and Plant Material. J. Funct. Foods.

[11] Huang B., Lei Y., Qin L., Liu J. (2012). Chemical Composition and Cytotoxic Activities of the Essential Oil from the Inflorescences of *Solidago canadensis* L., an Invasive Weed in Southeastern China. J. Essent. Oil Bear. Plants.

[12] Mishra D., Joshi S., Bisht G., Pilkhwal S. (2010). Chemical Composition and Antimicrobial Activity of *Solidago canadensis* Linn. Root Essential Oil. J. Basic Clin. Pharm..

[13] El-Sherei M., Khaleel A., Motaal A.A., Abd-Elbaki P. (2014). Effect of Seasonal Variation on the Composition of the Essential Oil of *Solidago canadensis* Cultivated in Egypt. J. Essent. Oil Bear. Plants.

[14] Council of Europe (2022). European Pharmacopoeia.

[15] Woźniak D., Ślusarczyk S., Domaradzki K., Dryś A., Matkowski A. (2018). Comparison of Polyphenol Profile and Antimutagenic and Antioxidant Activities in Two Species Used as Source of Solidaginis Herba-Goldenrod. Chem. Biodivers..

[16] Dobjanschi L., Păltinean R., Vlase L., Babotă M., Fritea L., Tămaş M. (2018). Comparative Phytochemical Research of *Solidago* Genus: *S. graminifolia*. Note I. Flavonoids. Acta Biol. Marisiensis.

[17] Dobjanschi L., Fritea L., Patay E.B., Tamas M. (2019). Comparative Study of the Morphological and Phytochemical Characterization of Romanian Solidago Species. Pak. J. Pharm. Sci..

[18] Thiem B., Wesołowska M., Skrzypczak L., Budzianowski J. (2001). Phenolic Compounds in Two *Solidago* L. Species from in Vitro Culture. Acta Pol. Pharm..

[19] Hrytsyk Y., Koshovyi O., Lepiku M., Jakštas V., Žvikas V., Matus T., Melnyk M., Grytsyk L., Raal A. (2024). Phytochemical and Pharmacological Research in Galenic Remedies of *Solidago canadensis* L. Herb. Phyton.

[20] Fursenco C., Calalb T., Uncu L., Dinu M., Ancuceanu R. (2020). *Solidago virgaurea* L.: A Review of Its Ethnomedicinal Uses, Phytochemistry, and Pharmacological Activities. Biomolecules.

[21] Azad M.A., Olawuni D., Kimbell G., Badruddoza A.Z.M., Hossain M.S., Sultana T. (2020). Polymers for Extrusion-Based 3D Printing of Pharmaceuticals: A Holistic Materials–Process Perspective. Pharmaceutics.

[22] El Aita I., Rahman J., Breitkreutz J., Quodbach J. (2020). 3D-Printing with Precise Layer-Wise Dose Adjustments for Paediatric Use via Pressure-Assisted Microsyringe Printing. Eur. J. Pharm. Biopharm..

[23] Kovalenko V.N. (2020). Compendium 2020. Medicines.

[24] Grodzinsky A.M. (1990). Medicinal Plants: Encyclopedic Guide.

[25] Fedotova V.V., Chelombytko V.A. (2012). Species of the Genus Solidago: Significance for Medical Practice, Study Prospects. Sci. Bull..

[26] Koshovyi O., Sepp J., Jakštas V., Žvikas V., Kireyev I., Karpun Y., Odyntsova V., Heinämäki J., Raal A. (2024). German Chamomile (*Matricaria chamomilla* L.) Flower Extract, Its Amino Acid Preparations and 3D-Printed Dosage Forms: Phytochemical, Pharmacological, Technological, and Molecular Docking Study. Int. J. Mol. Sci..

[27] Zagayko A.L., Kolisnyk T.Y., Chumak O.I., Ruban O.A., Koshovyi O.M. (2018). Evaluation of Anti-Obesity and Lipid-Lowering Properties of *Vaccinium Myrtillus* Leaves Powder Extract in a Hamster Model. J. Basic Clin. Physiol. Pharmacol..

[28] Kravchenko G., Krasilnikova O., Raal A., Mazen M., Chaika N., Kireyev I., Grytsyk A., Koshovyi O. (2022). *Arctostaphylos uva-ursi* L. Leaves Extract and Its Modified Cysteine Preparation for the Management of Insulin Resistance: Chemical Analysis and Bioactivity. Nat. Prod. Bioprospect..

[29] MacDougall C. (2004). Pharmacokinetics of Valaciclovir. J. Antimicrob. Chemother..

[30] Parfenov V.A. (2011). Use of L-Lysine Aescinate in Central Nervous System Diseases. Neurol. Neuropsychiatry Psychosom..

[31] Karpov Y.A. (2008). Perindopril Arginine: A New ACE Inhibitor Salt Increases Therapeutic Potential. Cardiovasc. Ther. Prev..

[32] Koshovyi O., Raal A., Kireyev I., Tryshchuk N., Ilina T., Romanenko Y., Kovalenko S.M., Bunyatyan N. (2021). Phytochemical and Psychotropic Research of Motherwort (*Leonurus cardiaca* L.) Modified Dry Extracts. Plants.

[33] Koshovyi O., Granica S., Piwowarski J.P., Stremoukhov O., Kostenko Y., Kravchenko G., Krasilnikova O., Zagayko A. (2021). Highbush Blueberry (*Vaccinium corymbosum* L.) Leaves Extract and Its Modified Arginine Preparation for the Management of Metabolic Syndrome—Chemical Analysis and Bioactivity in Rat Model. Nutrients.

[34] Koshovyi O., Vlasova I., Laur H., Kravchenko G., Krasilnikova O., Granica S., Piwowarski J.P., Heinämäki J., Raal A. (2023). Chemical Composition and Insulin-Resistance Activity of Arginine-Loaded American Cranberry (*Vaccinium Macrocarpon* Aiton, Ericaceae) Leaf Extracts. Pharmaceutics.

[35] Hrytsyk Y., Koshovyi O., Hrytsyk R., Raal A. (2024). Extracts of the Canadian Goldenrod (*Solidago canadensis* L.)—Promising Agents with Antimicrobial, Anti-Inflammatory and Hepatoprotective Activity. Sci. Pharm. Sci..

[36] Dobrochaeva D.N., Kotov M.I., Prokudin Y.N., Barbarich A.I. (1999). Key to Higher Plants of Ukraine.

[37] Vlasova I., Gontova T., Grytsyk L., Zhumashova G., Sayakova G., Boshkayeva A., Shanaida M., Koshovyi O. (2022). Determination of Standardization Parameters of *Oxycoccus macrocarpus* (Ait.) Pursh and Oxycoccus Palustris Pers. Leaves. Sci. Pharm. Sci..

[38] Kovaleva A.M., Georgievskyi G.V., Kovalev V.M., Komissarenko A.M., Timchenko M.M. (2002). Development of the Method of Standardization of the New Medicinal Product Piflamin. Pharmacom.

[39] Krivoruchko E., Markin A., Samoilova V.A., Ilina T., Koshovyi O. (2018). Research in the Chemical Composition of the Bark of Sorbus Aucuparia. Ceska A Slov. Farm..

[40] Vilkickyte G., Raudone L., Petrikaite V. (2020). Phenolic Fractions from *Vaccinium vitis-idaea* L. and Their Antioxidant and Anticancer Activities Assessment. Antioxidants.

[41] Uminska K., Gudžinskas Z., Ivanauskas L., Georgiyants V., Kozurak A., Skibytska M., Mykhailenko O. (2023). Amino Acid Profiling in Wild Chamaenerion Angustifolium Populations Applying Chemometric Analysis. J. Appl. Pharm. Sci..

[42] Olthof M.R., Hollman P.C.H., Buijsman M.N.C.P., Van Amelsvoort J.M.M., Katan M.B. (2003). Chlorogenic Acid, Quercetin-3-Rutinoside and Black Tea Phenols Are Extensively Metabolized in Humans. J. Nutr..

[43] Li J., Wang S.-P., Wang Y.-Q., Shi L., Zhang Z.-K., Dong F., Li H.-R., Zhang J.-Y., Man Y.-Q. (2021). Comparative Metabolism Study on Chlorogenic Acid, Cryptochlorogenic Acid and Neochlorogenic Acid Using UHPLC-Q-TOF MS Coupled with Network Pharmacology. Chin. J. Nat. Med..

[44] Alcázar Magaña A., Kamimura N., Soumyanath A., Stevens J.F., Maier C.S. (2021). Caffeoylquinic Acids: Chemistry, Biosynthesis, Occurrence, Analytical Challenges, and Bioactivity. Plant J..

[45] Zhao Y., Ren Y., Liu Z., Wang Z., Liu Y. (2022). The Metabolite Profiling of 3,4-dicaffeoylquinic Acid in Sprague–Dawley Rats Using Ultra-high Performance Liquid Chromatography Equipped with Linear Ion trap-Orbitrap MS. Biomed. Chromatogr..

[46] Rimon G., Sidhu R.S., Lauver D.A., Lee J.Y., Sharma N.P., Yuan C., Frieler R.A., Trievel R.C., Lucchesi B.R., Smith W.L. (2010). Coxibs Interfere with the Action of Aspirin by Binding Tightly to One Monomer of Cyclooxygenase-1. Proc. Natl. Acad. Sci. USA.

[47] Dong L., Yuan C., Orlando B.J., Malkowski M.G., Smith W.L. (2016). Fatty Acid Binding to the Allosteric Subunit of Cyclooxygenase-2 Relieves a Tonic Inhibition of the Catalytic Subunit. J. Biol. Chem..

[48] Stefanov O.V. (2001). Preclinical Studies of Drugs.

[49] The Law of Ukraine “On the Protection of Animals from Cruel Treatment” Dated 12/15/2009. https://zakon.rada.gov.ua/laws/show/3447-15#Text.

[50] (2009). On Approval of the Procedure for Preclinical Study of Medicinal Products and Examination of Materials of Preclinical Study of Medicinal Products.

[51] Council of the European Union (1999). European Convention for the Protection of Vertebrate Animals Used for Experimental and Other Scientific Purposes.

[52] Bergey D.H. (1957). Bergey’s Manual of Determinative Bacteriology.

[53] Kutsyk R.V. (2004). Screening Study of the Antimicrobial Activity of Medicinal Plants of the Carpathian Region against Polyantibiotic-Resistant Clinical Strains of Staphylococci. Message 1. Galician Med. Bull..

[54] Hrytsyk R.A., Kutsyk R.V., Yurchyshyn O.I., Struk O.A., Kireev I.V., Grytsyk A.R. (2021). The Investigation of Antimicrobial and Antifungal Activity of Some *Artemisia* L. Species. Pharmacia.

[55] Shanaida M., Oleschuk O., Lykhatskyi P., Kernychna I. (2017). Study of the Hepatoprotective Activity of the Liquid Extract of the Garden Thyme Herb in Tetrachloromethane Hepatitis. Pharm. J..

[56] Korobeinikova E.N. (1989). Modification of Determination of Lipid Peroxidation Products in the Reaction with Thiobarbituric Acid. Lab. Work.

[57] Viidik L., Seera D., Antikainen O., Kogermann K., Heinämäki J., Laidmäe I. (2019). 3D-Printability of Aqueous Poly(Ethylene Oxide) Gels. Eur. Polym. J..

[58] Koshovyi O., Heinämäki J., Raal A., Laidmäe I., Topelius N.S., Komisarenko M., Komissarenko A. (2023). Pharmaceutical 3D-Printing of Nanoemulsified Eucalypt Extracts and Their Antimicrobial Activity. Eur. J. Pharm. Sci..

[59] Riegel J., Mayer W., Havre Y.V. (2001). FreeCAD (Version 0.19.24291). http://www.freecad.org.

[60] Ukrainian Scientific Pharmacopoeial Center of Drugs Quality (2015). State Pharmacopoeia of Ukraine.

[61] Lapach S.N., Chubenko A.V., Babich P.N. (2000). Statistical Methods in Biomedical Research Using Excel.

[62] Zhu Q., Han Y., He Y., Fu Y., Yang H., Chen Y., Shi Y. (2023). Kaempferol Improves Breast Cancer-Related Depression through the COX-2/PGE2 Pathway. Front. Biosci..

[63] Khoswanto C. (2022). Molecular Docking Analysis of Quercetin and Diclofenac as Cox-2 Potential Inhibitors. J. Int. Dent. Med. Res..

[64] Bare Y., Krisnamurti G.C., Elizabeth A., Rachmad Y.T., Sari D.R.T., Wahyusari M.R., Lorenza G. (2019). The Potential Role of Caffeic Acid in Coffee as Cyclooxygenase-2 (COX-2) Inhibitor: In Silico Study. Biointerface Res. Appl. Chem..

[65] Mohalkar R., Poul B., Patil S.S., Hetkar M.A., Chavan S.D. (2014). A Review on Immediate Release Drug Delivery Systems. PharmaTutor.

[66] Kute V.G., Patil R.S., Kute V.G., Kaluse P.D. (2023). Immediate-release dosage form; focus on disintegrants use as a promising excipient. J. Drug Deliv. Ther..

